# Recognition patterns of acute kidney injury in hospitalized patients

**DOI:** 10.1093/ckj/sfae231

**Published:** 2024-07-22

**Authors:** Pasquale Esposito, Francesca Cappadona, Marita Marengo, Marco Fiorentino, Paolo Fabbrini, Alessandro Domenico Quercia, Francesco Garzotto, Giuseppe Castellano, Vincenzo Cantaluppi, Francesca Viazzi

**Affiliations:** Department of Internal Medicine, University of Genova, Genova, Italy; Division of Nephrology, Dialysis and Transplantation, IRCCS Ospedale Policlinico San Martino, Genova, Italy; Department of Internal Medicine, University of Genova, Genova, Italy; Division of Nephrology, Dialysis and Transplantation, IRCCS Ospedale Policlinico San Martino, Genova, Italy; Nephrology and Dialysis Unit, Department of Specialist Medicine, Azienda Sanitaria Locale CN1, Cuneo, Italy; Department of Precision and Regenerative Medicine and Ionian Area, Nephrology Dialysis and Transplantation Unit, University of Bari Aldo Moro, Bari, Italy; Nephrology and Dialysis Unit, ASST Nord Milano, Milan, Italy; Nephrology and Dialysis Unit, Department of Specialist Medicine, Azienda Sanitaria Locale CN1, Cuneo, Italy; Department of Cardiac Thoracic Vascular Sciences and Public Health, Unit of Biostatistics, Epidemiology and Public Health, University of Padua, Padua, Italy; Department of Nephrology, Dialysis and Renal Transplantation, Fondazione IRCCS Ca’ Granda Ospedale Maggiore Policlinico, Milan, Italy; Nephrology and Kidney Transplantation Unit, Department of Translational Medicine, SCDU Nefrologia e Trapianto Renale, University of Piemonte Orientale, Azienda Ospedaliero-Universitaria Maggiore della Carità, Novara, Italy; Department of Internal Medicine, University of Genova, Genova, Italy; Division of Nephrology, Dialysis and Transplantation, IRCCS Ospedale Policlinico San Martino, Genova, Italy

**Keywords:** administrative data, AKI, diagnosis, mortality, serum creatinine

## Abstract

**Background:**

Acute kidney injury (AKI) during hospitalization is associated with increased complications and mortality. Despite efforts to standardize AKI management, its recognition in clinical practice is limited.

**Methods:**

To assess and characterize different patterns of AKI diagnosis, we collected clinical data, serum creatinine (sCr) levels, comorbidities and outcomes from adult patients using the Hospital Discharge Form (HDF). AKI diagnosis was based on administrative data and according to Kidney Disease: Improving Global Outcomes (KDIGO) criteria by evaluating sCr variations during hospitalization. Additionally, patients were categorized based on the timing of AKI onset.

**Results:**

Among 56 820 patients, 42 900 (75.5%) had no AKI, 1893 (3.3%) had AKI diagnosed by sCr changes and coded in the HDF (full-AKI), 2529 (4.4%) had AKI reported on the HDF but not meeting sCr-based criteria (HDF-AKI) and 9498 (16.7%) had undetected AKI diagnosed by sCr changes but not coded in the HDF (KDIGO-AKI). Overall, AKI incidence was 24.5%, with a 68% undetection rate. Patients with KDIGO-AKI were younger and had a higher proportion of females, lower comorbidity burden, milder AKI stages, more frequent admissions to surgical wards and lower mortality compared with full-AKI patients. All AKI groups had worse outcomes than those without AKI, and AKI, even if undetected, was independently associated with mortality risk. Patients with AKI at admission had different profiles and better outcomes than those developing AKI later.

**Conclusions:**

AKI recognition in hospitalized patients is highly heterogeneous, with a significant prevalence of undetection. This variability may be affected by patients’ characteristics, AKI-related factors, diagnostic approaches and in-hospital patient management. AKI remains a major risk factor, emphasizing the importance of ensuring proper diagnosis for all patients.

KEY LEARNING POINTS **What was known:**

- Acute kidney injury (AKI) is a prevalent condition in hospitalized patients and is associated with elevated mortality and unfavourable outcomes.
- A timely and accurate diagnosis of AKI can significantly impact the clinical management and outcomes of hospitalized patients.
- Despite concerted efforts to standardize AKI diagnosis and raise awareness, clinical practice frequently fails to properly identify AKI.

**This study adds:**

- Recognition of AKI during hospitalization varies greatly, as there may be significant discrepancies between administrative and clinical data.
- Undetected AKI emerges as the predominant presentation pattern of hospital AKI.
- Although patients with undetected AKI may appear to have low-risk profiles, they experience higher mortality rates and more adverse clinical outcomes compared with those without AKI.

**Potential impact:**

- Methodologies relying on administrative data or solely on serum creatinine evaluation are unreliable for describing and characterizing the epidemiology of hospital-acquired AKI.
- Timely and accurate AKI diagnosis should be prioritized for all hospitalized patients, regardless of baseline patient characteristics, comorbidities and required intensity of care.
- The implementation of educational programs, along with exploration of new biomarkers and technologies, should be investigated to improve AKI diagnosis and outcomes.

## INTRODUCTION

Acute kidney injury (AKI) is a common complication among hospitalized patients with significant clinical consequences [1]. Short-term effects include increased in-hospital mortality, length of hospitalization and costs [2]. Moreover, it is widely recognized that an AKI episode is an independent risk factor for subsequent acute injury and also contributes to the development of chronic kidney disease (CKD) [3].

Hence the implementation of strategies aimed at preventing, promptly identifying and treating AKI has become of paramount importance. The primary step toward enhancing AKI-related outcomes involves the establishment of clear indications for AKI diagnosis and risk assessment [4].

In this context, the introduction of Kidney Disease: Improving Global Outcomes (KDIGO) criteria in 2012 has represented the most notable endeavour to standardize AKI diagnosis and management [5, 6]. Nonetheless, even if there is a widespread application of these criteria in epidemiological and clinical studies, in real-life scenarios the actual incidence of AKI continues to be substantially underestimated.

A comprehensive meta-analysis by Susantitaphong *et al.* [7], including 312 studies published after 2004, reported a global in-hospital AKI incidence of 10.7%, which increased to 23.2% when analysing 154 studies using the KDIGO classification criteria. Strikingly, when the AKI diagnosis relied solely on administrative code data, the incidence dropped to 2.9%, highlighting a profound underestimation of this condition. Moreover, a multicentre survey conducted in China revealed that when defining AKI by both standard and extended KDIGO criteria its prevalence was ≈3%, but only 25.8% of AKI cases were formally identified and documented within hospital records [8]. Notably, factors contributing to the underrecognition of AKI included patients originating from poor regions, milder AKI stages and the absence of nephrologist consultations.

Beyond the epidemiological aspects of AKI recognition, there is an ongoing debate regarding its clinical implications. In 2013, before the adoption of KDIGO criteria, Wilson *et al*. [9] observed that patients with recorded AKI exhibited improved survival compared with those without formally documented AKI. The authors attributed this improved outcome to the fact that patients with a confirmed AKI diagnosis received more frequent nephrology consultations (31% versus 6%; *P* < .001) and thus more specific treatments. More recently, Wu *et al*. [10], in a retrospective observational study using propensity matching, compared 241 patients with unrecognized AKI with 241 patients with promptly recognized AKI. Surprisingly, in this instance, the underrecognition of AKI did not impact all-cause mortality. Nonetheless, despite the presence of epidemiological reports and studies in controlled settings, data on the extent and clinical correlates of AKI recognition in actual clinical scenarios remain scarce. To address this issue, we conducted a large cohort study to characterize the AKI recognition patterns among hospitalized patients.

## MATERIALS AND METHODS

A retrospective observational study was conducted at our university hospital. We enrolled adult patients during their first hospital admission from 1 January 2016 to 31 December 2019.

Patients with chronic kidney disease (CKD) stages 4–5 or those undergoing maintenance renal replacement therapy, identified through the International Classification of Disease, 9th Revision, Clinical Modification (ICD-9-CM) diagnosis codes on the Hospital Discharge Form (HDF), were excluded from the research algorithm. The study protocol was approved by our institutional review board (registration number CER Liguria: 515/2020) and the requirement for informed consent was waived. The study adhered to the principles outlined in the Helsinki Declaration.

### Data collection

We retrieved all data from the hospital's electronic database, including clinical information, comorbidities, serum creatinine (sCr) levels, duration of hospitalization, mortality and type of discharge. Patients with a minimum of two sCr determinations were included in the study. Comorbidities were identified using all ICD-9-CM codes listed on the HDF [11]. sCr levels were recorded upon admission, discharge and at the lowest and highest (peak) points during hospitalization.

### Definitions

**Figure 1:**
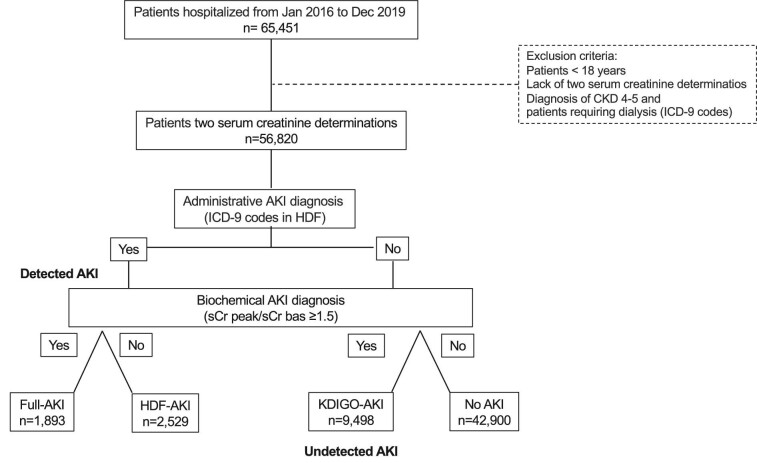
Selection strategy for the definition of AKI recognition patterns among patients hospitalized during the study period (2016–2019).

We identified patients developing AKI using two different approaches. The first method relied on administrative data reported on the HDF (codes 584.5–584.9) and the second method was a biochemical diagnosis based on changes in sCr levels collected during hospitalization. For the latter, we applied an ‘extended’ KDIGO criteria [12]. We used the lowest sCr level as the baseline value and we analysed the ratio between the maximum and minimum values. AKI was defined by a ratio ≥1.5. Subsequently, AKI was classified into stages 1, 2 and 3 according to the KDIGO framework, corresponding to 1.5–1.9 times, 2–2.9 times and ≥3 times the baseline creatinine. Urine output was not considered due to limited available data. Based on the two AKI definition approaches, we defined ‘detected’ AKI as when the AKI diagnosis code was found on the HDF and ‘undetected’ as when a formal diagnosis of AKI was lacking. Merging these two definitions, our cohort was categorized into four groups: patients with both an administrative and sCr-based AKI diagnosis (full-AKI), patients with only detected administrative AKI not meeting KDIGO criteria (HDF-AKI), patients meeting the KDIGO criteria but with undetected AKI (KDIGO-AKI) and patients without AKI (no-AKI) (Fig. 1).

Moreover, among all the patients meeting the KDIGO criteria for AKI diagnosis, we further distinguished between patients presenting peak sCr within 48 h from hospital admission (adm-AKI) and those presenting peak sCr later [in-hospital AKI (IH-AKI)] [13].

The estimated glomerular filtration rate (eGFR) was calculated using the Chronic Kidney Disease Epidemiology Collaboration creatinine-based equation [14].

Finally, considering the hospital wards of the patients, we incorporated the different departments into the following categories: ‘medical ward’, which includes internal medicine and specialty medicine wards; ‘surgery’, encompassing all surgery departments; ‘intensive care unit’ (ICU), covering general ICU, cardiac surgical and neurosurgical ICUs; and ‘emergency medicine’, comprising the wards directly associated with the Emergency Department.

### Outcomes

The primary objective of this study was to determine the incidence of different AKI recognition patterns during hospitalization. Subsequently we assessed factors associated with AKI undetection and compared the clinical outcomes, including mortality, length of hospital stay (LOS) and type of discharge, in different AKI recognition groups.

### Statistical analysis

Normally distributed variables are presented as mean ± standard deviation (SD) and were compared using independent or paired *t*-tests as appropriate. Group comparisons were conducted through analysis of variance. Proportions were compared using the χ^2^ test or Fisher's exact test when applicable. The incidence rate of AKI was calculated and odds ratios (ORs) with 95% confidence intervals (CIs) were computed using logistic regression coefficients. Time-to-event analyses were conducted employing the following methods: Kaplan–Meier method for survival curve estimation and logrank test for comparisons and univariate and multivariate Cox regression models, with risk reported as hazard ratios (HRs) along with 95% CIs. All clinically plausible clinical variables were included as covariates. The time variable was defined as the interval between the baseline date and the date of endpoint occurrence until day 30 of hospitalization. Logistic regression was used to analyse risks and expressed as ORs and their 95% CIs. Post hoc analyses among groups were made by Bonferroni test.

Statistical calculations were performed using Stata version 14.2 (StataCorp, College Station, TX, USA). The null hypothesis was rejected for *P*-values <.05.

## RESULTS

### Patient characteristics

We included a total of 56 820 patients (52.2% females) with an average age of 70.1 ± 18.7 years. According to the ICD-9-CM codes, 8.6% of these patients had diabetes, 9.3% had heart failure (HF), 12.9% had cancer and 8% had previous CKD. Myocardial ischaemia was reported in 2.5% of patients, while sepsis was seen in 3.9% of the total population. Upon admission, the mean sCr level was 1.12 ± 0.98 mg/dl and the eGFR was 74.7 ± 29.2 ml/min/1.73 m^2^. The majority of patients were admitted to medical wards, with 2% requiring admission to the ICU (see Table 1).

**Table 1:** Main clinical characteristics of patients hospitalized in the studied period (January 2016–December 2019), distinguished according to the patterns of AKI detection.

Characteristics	All patients (*N* = 56 820)	No-AKI^a^ [*n* = 42 900 (75.5%)]	Full-AKI [*n* = 1893 (3.3%)]	HDF-AKI [*n* = 2529 (4.4%)]	KDIGO-AKI [*n* = 9498 (16.7%)]	*P*-value, full-AKI versus HDF-AKI	*P*-value, full-AKI versus KDIGO-AKI
Female, *n* (%)	29 653 (52.2)	22 274 (51.9)	934 (49.3)	1189 (47)	5256 (55.4)	0.75	<0.001
Age (years), mean ± SD	70.1 ± 18.7	67.7 ± 19.5	78.7 ± 11.8	79.2 ± 12.5	76.7 ± 14.2	0.8	<0.001
CKD, *n* (%)	4526 (8.0)	2919 (6.8)	256 (13.5)	335 (13.2)	1016 (10.7)	1	0.001
Diabetes mellitus, *n* (%)	4877 (8.6)	3335 (7.8)	292 (15.4)	482 (19)	768 (8.1)	0.001	<0.001
Heart failure, *n* (%)	5237 (9.3)	2963 (6.9)	366 (19.3)	486 (19.2)	1422 (15.0)	1	<0.001
Neoplasia, *n* (%)	7315 (12.9)	5185 (12.1)	254 (13.4)	290 (11.4)	1586 (16.7)	0.3	0.001
Acute myocardial ischaemia, *n* (%)	1393 (2.5)	924 (2.1)	65 (3.4)	76 (3)	328 (3.4)	1	1
Sepsis, *n* (%)	2229 (3.9)	744 (1.7)	269 (14.2)	193 (7.6)	1023 (10.8)	<0.001	<0.001
Admission sCr (mg/dl), mean ± SD	1.14 ± 0.89	0.98 ± 0.57	2.48 ± 1.8	1.88 ± 1. 7	1.37 ± 1.03	<0.001	<0.001
Peak sCr (days), day from admission, median (IQR)	–	–	2 (1–8)	1 (1–3)	3 (1–11)	<0.001	<0.001
sCr variation (peak/lowest) (mg/dl), mean ± SD			2.84 ± 1.83	1.17 ± 0.16	2.2 ± 1.2	<0.001	<0.001
AKI stage (*N* = 11 381), *n* (%) 1 2 3			738 (39) 586 (31) 569 (30)		5341 (56.2) 2667 (28.2) 1480 (15.6)	–	<0.001
Ward (patients), *n* (%)						OverallAKI^b^, *n* (%)	AKI undetection (%)^c^
Medical	34 093 (60.0)	25 528 (59.5)	1188 (62.6)	1122 (44.3)	6258 (65.9)	8568 (25)	73
Surgical	12 694 (22.3)	10 133 (23.6)	122 (6.4)	217 (8.6)	2222 (23.4)	2561 (20.1)	86
Emergency Department	8876 (15.6)	6767 (15.8)	478 (25.2)	1167 (46.1)	464 (4.9)	2109 (23.7)	22
ICU	1154 (2.0)	472 (1.1)	105 (5.5)	23 (0.9)	554 (5.8)	682 (59)	81
Total						13 920 (24.5)	68.2

^a^ *P* < .001 for all AKI groups versus no-AKI (except for incidence of neoplasia versus HDF-AKI).

^b^Overall AKI incidence included AKI diagnosed according to administrative documentation and/or biochemical criteria (i.e. full-AKI + HDF-AKI + KDIGO-AKI).

^c^AKI undetection incidence refers to AKI diagnosed by sCr but not formally reported (i.e. KDIGO-AKI/overall AKI).

### Patterns of AKI detection during hospitalization

**Figure 2:**
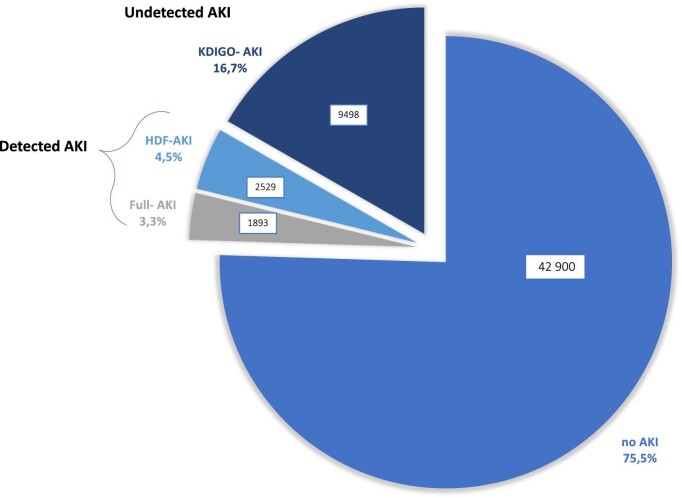
AKI pattern distribution among patients hospitalized during the study period (2016–2019).

Following the methodology previously described, we classified our patient population into four distinct groups: 42 900 patients (75.5%) without AKI (no-AKI), 1893 patients (3.3%) with detected AKI diagnosis formally documented and meeting KDIGO criteria for AKI (full-AKI), 2529 patients (4.4%) with detected AKI but not meeting sCr-based criteria for AKI (HDF-AKI) and 9498 patients (16.7%) diagnosed with AKI as determined by sCr changes but remaining undetected, i.e. without a formal documentation (KDIGO-AKI). (Fig. 2).

Taking both administrative and biochemical criteria into account, the overall incidence of AKI was 24.5% (*n* = 13 920 patients), with a higher incidence observed in the ICU (59%).

Compared with no-AKI patients, all patients with AKI were older and had a higher prevalence of comorbidities, myocardial ischaemia and sepsis (Table 1). Overall, upon analysing the various patterns of AKI recognition, we observed that only a minority of AKI episodes (31.8%) were officially documented, resulting in an undetection rate of 68.2%.

Furthermore, AKI undetection was observed across all hospital departments, albeit with varying incidence rates among different wards. Interestingly, patients admitted to the Emergency Department exhibited a lower rate of AKI undetection compared with other wards.

A comprehensive analysis of the different AKI patient groups revealed distinct characteristics.

Patients in the HDF-AKI cohort, compared with the full-AKI group, had similar general characteristics but presented with lower sCr levels at admission, with smaller sCr changes during hospitalization. Interestingly, a higher percentage of these patients were admitted to the Emergency Department (46.1% versus 25.2%; *P* < .001).

Analysing KDIGO-AKI patients, in whom AKI diagnosis was formally undetected, we found that these patients were significantly younger, had a higher proportion of females and exhibited a lower prevalence of comorbidities such as diabetes, CKD and HF. Moreover, their sCr levels at admission were significantly lower and they were more often admitted to surgery wards.

Lastly, compared with full-AKI patients, those with KDIGO-AKI developed AKI later during the hospitalization and displayed a higher incidence of stage 1 AKI, with a lower incidence of stages 2–3.

### Factors associated with AKI undetection

To assess the clinical factors associated with the risk of AKI undetection, we focused our analysis solely on patients meeting KDIGO criteria for AKI (i.e. the full-AKI and KDIGO-AKI groups).

In the multivariate model, we noted that male sex, older age, diabetes, AKI at hospital admission and higher AKI stages were associated with a decreased risk of undetection. Conversely, a longer LOS and particularly admission to a surgical ward were associated with an increased risk of AKI undetection (Table 2).

**Table 2:** Logistic models for AKI undetection in hospitalized patients developing AKI (diagnosed according to sCr changes following KDIGO criteria).

	Univariate			Multivariate		
Risk factors	OR	95% CI	*P*-value	OR	95% CI	*P*-value
Gender (male)	0.78	0.71–0.86	<.0001	0.73	0.66–0.81	<.0001
Age	0.98	0.98–0.99	<.0001	0.98	0.98–0.99	<.0001
Comorbidities						
Heart failure	0.82	0.71–0.95	<.0001	0.90	0.78–1.02	.117
Diabetes	0.48	0.41–0.55	<.0001	0.47	0.40–0.55	<.0001
Admission AKI	0.67	0.60–0.74	<.0001	0.68	0.61–0.76	<.0001
AKI stage	0.60	0.56–0.64	<.0001	0.54	0.50–0.57	<.0001
LOS (days)	1.01	1.01–1.015	<.0001	1.01	1.01–1.012	<.0001
Surgical ward	3.8	3.2–4.5	<.0001	3.5	297–4.2	<.0001

### Outcomes according to the AKI detection pattern

Among the AKI groups, full-AKI patients showed the highest mortality rate, but both the HDF-AKI and KDIGO-AKI groups had a significantly higher mortality compared with the no-AKI patients (Table 3). Additionally, patients with undetected KDIGO-AKI presented a longer LOS and a greater proportion of them required a protected discharge, indicating a need for continued care or support post-hospitalization.

**Table 3:** Clinical outcomes of the entire population of hospitalized patients (2016–2019) according to AKI recognition.

Characteristics	All patients	No-AKI	Full- AKI	HDF-AKI	KDIGO-AKI	*P*-value, full-AKI versus HDF-AKI	*P*-value, full-AKI versus KDIGO-AKI
Patients, *n*	56 820	42 900	1893	2529	9498		
In-hospital outcomes							
Mortality rate, *n* (%)	4720 (8.3)	1636 (3.8)	578 (30.5)	504 (19.9)	2 002 (21.1)	<.0001	<.0001
LOS (days), mean ± SD	12.1 ± 12.5	9.5 ± 8.6	20 ± 2	8.3 ± 8.2	23.4 ± 18.5	<.0001	<.0001
Discharge status						<.0001	<.0001
At home, *n* (%)	40 847 (71.9)	33 417 (77.9)	951 (72.3)	1612 (79.6)	4867 (51.2)		
Protected, *n* (%)	11 253 (21.6)	7946 (19)	364 (27.7)	413 (20.4)	2629 (35)		
sCr (mg/dl) at discharge, mean ± SD	1.08 ± 0.77	0.97 ± 0.54	1.9 ± 1.41	1.7 ± 1.46	1.22 ± 0.94	<.0001	<.0001

Mortality risk factors were assessed for the entire study population using both univariate and multivariate Cox analyses. Univariate analysis revealed that factors such as age, male sex, acute events like myocardial ischaemia and sepsis, neoplasia, admission sCr levels, ICU admission and the occurrence of AKI were all significantly associated with the mortality rate. In the multivariate analysis, even after adjusting for clinical and demographic factors, both global AKI (i.e. AKI diagnosed according to administrative documentation and/or biochemical criteria) and undetected AKI remained significantly and independently associated with mortality, even when assessed separately (Table 4) (models 1 and 2).

**Table 4:** Univariate and multivariate Cox regression analyses of in-hospital mortality in hospitalized patients.

	Univariate			Multivariate model 1			Multivariate model 2		
Characteristics	HR	95% CI	*P*-value	HR	95% CI	*P*-value	HR	95% CI	*P*-value
Gender (male)	1.08	1.02–1.14	0.11	1.13	1.06–1.20	<.0001	1.17	1.09–1.25	<.0001
Age	1.04	1.04–1.04	<.0001	1.04	1.04–1.05	<.0001	1.04	1.04–1.05	<.0001
Acute illness									
Acute myocardial ischaemia	2.05	1.80–2.34	<.0001	1.43	1.25–1.64	<.0001	1.34	1.14–1.56	<.0001
Sepsis	2.62	2.43–2.82	<.0001	2.29	2.11–2.47	<.0001	2.14	1.95–2.35	<.0001
Neoplasia	1.33	1.23–1.42	<.0001	1.75	1.62–1.88	<.0001	1.94	1.79–2.10	<.0001
Admission sCr	1.19	1.17–1.20	<.0001	1.14	1.12–1.16	<.0001	1.15	1.12–1.18	<.0001
ICU stay	1.88	1.74–2.04	<.0001	2.43	2.23–2.69	<.0001	2.8	2.55–3.06	<.0001
Overall AKI^a^	2.65	2.49–2.82	<.0001	1.69	1.58–1.81	<.0001			
Undetected AKI^b^	1.91	1.77–2.05	<.0001				1.27	1.17–1.36	<.0001

^a^Overall AKI included AKI diagnosed according to administrative documentation and/or biochemical criteria.

^b^Undetected AKI refers to AKI diagnosed on sCr but not formally reported.

**Figure 3:**
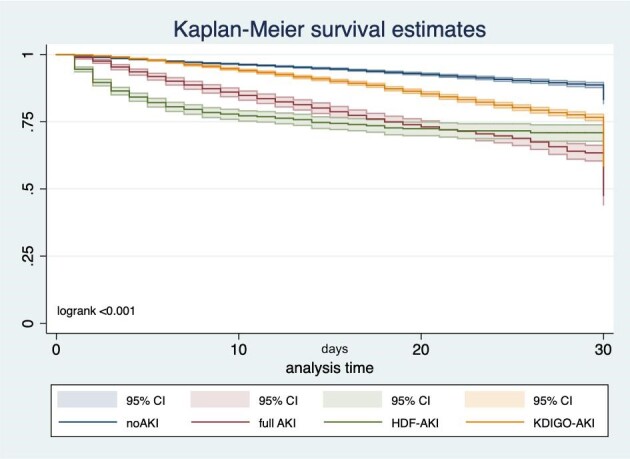
30-day Kaplan–Meier survival estimation based on AKI recognition patterns.

Furthermore, 30-day Kaplan–Meier analysis of intrahospital survival showed distinct mortality time profiles among the various AKI recognition groups. While early mortality was higher in the HDF-AKI group, mortality increased later in the full-AKI patients. Notably, even patients in the KDIGO-AKI group had significantly higher mortality compared with those without AKI (logrank <0.001; Fig. 3).

### Admission AKI

To further characterize AKI presentation and outcomes in our population, we focused on patients diagnosed with AKI based on sCr changes (*n* = 11 391), distinguishing them between those with peak sCr within 48 h from hospital admission (adm-AKI) and those with peak sCr later (IH-AKI) (Table 5).

**Table 5:** Main clinical characteristics of patients with AKI diagnosis based on KDIGO criteria according to the timing of AKI onset.

Characteristics	Adm-AKI [*n* = 5438 (47%)]	IH-AKI [*n* = 5953 (53%)]	*P*-value
Female, *n* (%)	3041 (55.9)	3190 (52.9)	.001
Age (years), mean ± SD	76.7 ± 14.4	77.3 ± 13.2	.041
CKD, *n* (%)	641 (11.8)	710(11.9)	.835
Diabetes mellitus, *n* (%)	503 (9.2)	557 (9.4)	.8
Heart failure, *n* (%)	705 (13)	1083 (18.1)	<.001
Neoplasia, *n* (%)	798 (14.7)	1042 (17.5)	<.001
Acute myocardial ischaemia, *n* (%)	143 (2.6)	250 (4.2)	<.001
Sepsis, *n* (%)	543 (0.9)	749 (12.6)	<.001
Admission sCr (mg/dl), mean ± SD	1.89 ± 1.5	1.25 ± 0.91	<.001
Peak sCr (day), day from admission, median (IQR)	1 (1–1)	10 (5–18)	<.001
AKI stage, *n* (%) 1 2 3	2980 (54.8) 1611 (29.6) 847 (15.6)	3099 (52.1) 1652 (27.7) 1202 (20.2)	<.001
Ward patients, *n* (%)			<.001
Medical	3399 (62.5)	4045 (67.9)	
Surgical	1138 (20.9)	1206 (20.2)	
Emergency Department	662 (12.2)	280 (4.7)	
ICU	238 (4.4)	421 (7)	

The adm-AKI group comprised 5438 patients (47%) who, compared with IH-AKI patients, were significantly younger, had a higher proportion of females and had a lower burden of comorbidities. Additionally, adm-AKI patients exhibited a lower incidence of stage 3 AKI and were more frequently admitted to the Emergency Department, with fewer ICU admissions. Concerning outcomes, adm-AKI patients had lower mortality, shorter LOSs and lower sCr levels at discharge (Table 6).

**Table 6:** Clinical outcomes of patients with AKI diagnosis based on KDIGO criteria according to the timing of AKI onset.

Characteristics	Adm-AKI	IH-AKI	*P*-value
Patients, *n*	5438	5953	
In-hospital outcomes			
Mortality rate, *n* (%)	751 (13.8)	1829 (30.7)	<.001
LOS (days), mean± SD	18.7 ± 15	26.6 ± 20.56	<.001
Discharge status			<.001
At home, *n* (%)	3221 (59.2)	2597 (43.6)	
-Protected, *n* (%)	1466 (30)	1527 (25.6)	
sCr (mg/dl) at discharge, mean ± SD	1.08 ± 0.69	1.5 ± 1.2	<.001

**Figure 4:**
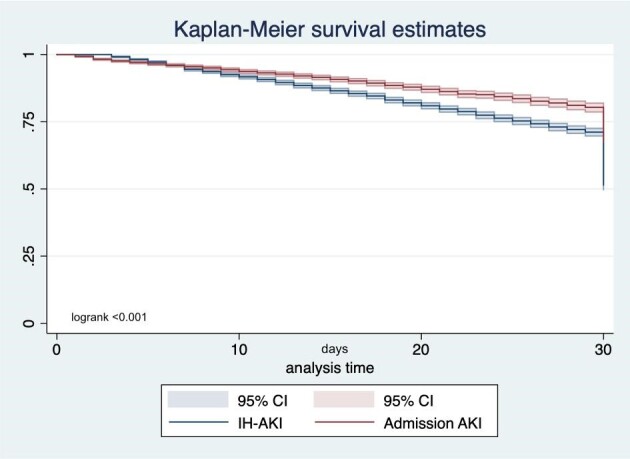
30-day Kaplan–Meier survival estimation based on timing of AKI onset. Only patients with biochemical diagnosis of AKI based on sCr changes (*n* = 11 391) were included in this analysis.

However, Kaplan–Meier analysis revealed temporal variations in mortality rates between the two groups. Specifically, although overall mortality was significantly higher in the IH-AKI patients, early mortality rates were nearly equivalent between the two groups (Fig. 4).

## DISCUSSION

The primary purpose of this study was to investigate and characterize the different patterns of AKI recognition in a large population of hospitalized patients. Thus we evaluated two different approaches to AKI diagnosis, one based on administrative data and the other based on sCr values.

Comparison of these two strategies allowed us to highlight the great heterogeneity of AKI recognition in real-world scenarios. First, we observed that AKI undetection, i.e. the occurrence of AKI episodes not formally documented, remains a substantial problem. Indeed, even though our cohort exhibited a total AKI incidence similar to that reported in global AKI epidemiological studies (24.5%) [15], we found that AKI was undetected in a significant two-thirds of cases. This observation aligns with previous reports conducted in other countries and settings and raises several important considerations [8, 16, 17]. Due to the considerable clinical, social and economic implications of AKI, extensive research and collaborative initiatives have been dedicated to exploring AKI epidemiology and standardizing its definition and diagnosis criteria [18, 19].

These efforts have undoubtedly improved our understanding of AKI pathophysiology and promoted knowledge and data sharing [20].

Nevertheless, real-world data, such as those presented here, indicate that these efforts are still falling short in ensuring accurate and proper AKI detection, which is a crucial step in enhancing AKI prevention and management [21]. Notably, a high rate of AKI undetection was observed in all hospital wards, including the ICU, where patients are at high risk for AKI development [22].

Furthermore, the substantial disparity between administrative data and AKI diagnoses based on sCr evaluations suggests that information obtained from administrative sources should be interpreted cautiously and may not be adequate for reliably studying in-hospital AKI epidemiology [23]. Previous research has indicated that factors such as a history of CKD, the severity of AKI and regional economic conditions can impact the recognition of AKI [8, 15]. Within our cohort, patients with undetected AKI exhibited distinct traits: they were younger, had a lower prevalence of CKD history, experienced milder forms of AKI (with a higher incidence of stage 1 AKI) and were predominantly admitted to surgical wards compared with those with a formal diagnosis. Notably, the undetection of AKI was more prevalent among female patients.

Multivariate analysis identified a lower burden of comorbidities, female sex, younger age, AKI occurrence late during the hospitalization, mild AKI severity, longer LOS and admission to a surgical department as independent risk factors for the undetection of AKI. A plausible explanation for this observation may be the limited attention given in the hospital setting to monitoring kidney function in these specific patient profiles, potentially leading to an incorrect perception of them as being at low risk for developing AKI.

Consistent with this hypothesis, a study by Aitken *et al*. [24], focusing on the quality of care provided to AKI patients in a cohort of >1500 hospitalized patients, found that clinician inexperience and inadequate clinical review and investigations were the main causes of delayed AKI diagnosis or non-recognition.

Furthermore, it is important to highlight the high prevalence of AKI undetection in surgical departments. This finding is consistent with previous studies that have demonstrated considerable variability in the incidence of post-surgical AKI, which remains largely underdiagnosed [25–27]. Additionally, it should be noted that the comparison between administrative and biochemical AKI diagnoses revealed discrepancies even among patients formally diagnosed with AKI in hospital documentation. Specifically, we observed that the majority of these patients did not meet the sCr-based KDIGO criteria for an AKI diagnosis.

The reasons behind this discrepancy remain unclear. In the absence of precise data, we can only speculate that, aside from coding errors, AKI in these patients may have developed prior to hospital admission, potentially indicating cases of community-acquired AKI (CA-AKI). Alternatively, the diagnosis of AKI in these cases might have been based on urine output assessment rather than changes in creatinine levels, a possibility particularly relevant in elderly or malnourished individuals, where creatinine values may remain low due to reduced muscle mass [28].

These considerations are crucial as they underscore the potential limitations of AKI diagnosis based solely on in-hospital creatinine evaluation.

Looking at clinical outcomes, we found that all patients with AKI had worse outcomes compared with patients without AKI. Moreover, multivariate analysis demonstrated that AKI is an independent predictor of mortality. However, disparities were noted among the different AKI recognition groups.

Patients in the full-AKI group demonstrated the highest in-hospital mortality rate, while those in the administrative group had higher mortality during the initial days of hospitalization. Interestingly, patients in the KDIGO-AKI group, despite experiencing milder forms of AKI, also exhibited higher mortality compared with patients without AKI. Additionally, discharge conditions were worse for these patients, and they exhibited poorer kidney function at discharge, suggesting a potentially heightened risk of AKI recurrence or progression to CKD.

Beyond diagnosis, another element that may impact clinical presentations and outcomes is the timing of AKI onset. To explore this peculiar aspect, aiming to minimize possible confounding factors, we focused solely on patients with a biochemical sCr-based AKI diagnosis.

Consistent with previous findings, we found significant differences between patients who presented with AKI at admission and those who met AKI criteria later [29]. Patients with AKI at admission were younger, had fewer comorbidities and experienced milder AKI stages, resulting in better outcomes compared with those with in-hospital AKI onset. In addition, it is likely that part of this cohort developed AKI before hospitalization, falling within the definition of CA-AKI.

The substantial differences between these patient groups underscore the importance of considering the timing of AKI onset as a crucial variable in AKI management, highlighting the utility of pre-hospitalization data availability.

A common finding, further sustained by our data, is that appropriate and early AKI diagnosis remains insufficient [30]. To address this issue, numerous research efforts have been conducted to evaluate the effectiveness of various biomarkers and digital tools [31, 32].

These tools encompass various technologies and applications, including computing platforms, connectivity, software, hardware and sensors [33]. The final goal of these techniques is to generate e-alert systems as part of a clinical decision support system (CDSS). E-alert systems are increasingly being implemented across hospital settings with various aims, including the prevention of nephrotoxicity and early detection of new episodes of sepsis in the ICU [34–36].

Previous experiences with e-alert systems in AKI management have yielded mixed results. One critical challenge is the heterogeneity of the systems implemented. Although e-alerts are generally triggered by detecting changes in serum creatinine, the threshold for alarms and the contribution of other elements, such as urine output or clinical and demographic data, are still to be defined [37].

Moreover, even when early detection is possible, implementing automatic alerts does not necessarily lead to improved clinical outcomes [38]. This may be because many e-alert-based CDSSs were designed for AKI recognition rather than for facilitating active clinical interventions [39]. Thus a simple AKI diagnosis might not be sufficient to improve outcomes without a well-defined and suitable response plan. Moreover, the widespread adoption of new technologies presents some limitations, including high costs, privacy concerns and equity, especially in terms of accessibility in low-income countries [40]. So while novel biomarkers and technologies hold promise, their validation and adoption in the general population are still pending [41].

Our study has several limitations. First, its retrospective, observational, single-centre design may restrict the generalizability of findings due to potential variations in AKI incidence, mortality rates and procedures across different hospitals, regions and countries [42].

Furthermore, although our results are consistent with those reported in large epidemiological studies, the pragmatic method used for calculating AKI, while reflective of real-world clinical practices, introduces a potential source of variability. Specifically, the choice of baseline sCr, and notably the absence of data on urine output, may significantly impact the accuracy of AKI incidence assessments and recognition [43].

In particular, the lack of evaluation of urine output represents a significant limitation not only of our study, but also for much of the current literature on AKI epidemiology [8, 44].

Additionally, we have no data on specific causes for hospitalization, such as AKI aetiology, which could influence disease presentation and outcomes.

Lastly, comorbidities were identified using administrative codes entered in the hospital database, introducing the possibility of information bias.

In conclusion, our study confirming AKI as an independent risk condition highlights the significant heterogeneity of AKI recognition within the hospital setting. This variability is influenced by various clinical factors, including patient characteristics, AKI-related factors (such as severity and timing of onset) and in-hospital patient management. However, it may also result from the potential limitations of current diagnosis approaches in fully elucidating in-hospital AKI epidemiology.

Furthermore, we found that even a large subset of patients apparently at low risk and developing milder forms of AKI, who are at increased risk of AKI undetection, experience adverse outcomes. So while our data may not establish a direct correlation between recognition patterns and outcomes, we advocate for timely and accurate recognition of AKI as a priority for all hospitalized patients.

A more comprehensive methodology may require integrating various sources of information, including administrative data, biochemical alterations, biomarkers and accurate urine output measurements, collected both within and outside the hospital setting [45]. This approach could be addressed to the general hospitalized population or focused on subjects at higher risk of AKI undetection, such as surgical patients [46].

In this context, while innovative biomarkers and technologies are advancing toward practical application, raising awareness about AKI among all healthcare professionals, investing in education and promoting clinical and scientific collaborations remain crucial interventions [47]. Whether and how early and appropriate AKI diagnosis can translate into effective actions to improve patient outcomes remains to be verified in prospectively designed trials.

## Data Availability

The data underlying this article will be shared upon reasonable request to the corresponding author.
